# Femtosecond Laser Two-Photon Absorption for Simulating Single-Event Effects and Defining the Safe Operating Area of SiC Power MOSFETs

**DOI:** 10.3390/mi17080894

**Published:** 2026-07-26

**Authors:** Chenguang Zhang, Hong Yin, Liang Shi, Xuan Wen, Zheng Ma, Hanwu Jia

**Affiliations:** National Key Laboratory on Vacuum Technology and Physics, Lanzhou Institute of Physics, Lanzhou 730000, China; zhangcg0319@163.com (C.Z.)

**Keywords:** silicon carbide, power MOSFET, femtosecond laser, two-photon absorption, single-event effects, equivalent linear energy transfer, safe operating area

## Abstract

Single-event burnout (SEB) remains a persistent threat to SiC power MOSFETs in space, yet rapid evaluation of SEB susceptibility without costly heavy-ion campaigns is challenging. This work demonstrates that femtosecond laser two-photon absorption (TPA) can fill that role for a commercial 1200 V SiC MOSFET—provided the laser energy is correctly mapped to heavy-ion linear energy transfer (LET). We derive an equivalent LET model that incorporates the thermal spike effect, giving LET_eq = Γ_1_E_0_^2^ + Γ_2_E_0_^4^, which corrects the classical square law at high excitation intensities where it fails. Three ionization-driven failure signatures emerge: drain-to-gate and drain-to-source single-event leakage current (SELC), and SEB. The SEB threshold saturates near 500 V once LET exceeds 25 MeV·cm^2^/mg—roughly 42% of the device’s 1200 V rating. From these thresholds, we define a safe operating area: below 200 V is safe, 200–600 V risks SELC degradation, and above 600 V carries high SEB risk. Benchmarking against published heavy-ion data shows SEB threshold agreement within 15%, and within 5% at high LET. We stress that the TPA method captures ionization-driven effects only; it does not replicate displacement damage. These results support rapid, laser-based screening of SiC power devices for radiation hardness.

## 1. Introduction

Single-event burnout (SEB) has emerged as a leading reliability concern for SiC power MOSFETs deployed in space. Unlike gradual parametric drift, SEB produces a catastrophic short between source and drain—often in a single event. Galactic cosmic rays, with their broad energy spectrum and high linear energy transfer (LET), are the primary culprit [1,2]. The increasing voltage ratings of SiC devices for aerospace power systems demand a rapid and reliable method for evaluating SEB susceptibility [3].

The established approach—heavy-ion broad-beam testing—is effective but painfully slow and expensive. Beam time at accelerator facilities is oversubscribed, test campaigns stretch for months, and the cost per data point is substantial [4]. Pulsed-laser techniques have long offered a complementary route for silicon devices [5], but translating them to wide-bandgap semiconductors is not straightforward. SiC’s 3.2 eV bandgap demands single-photon energies in the ultraviolet for conventional absorption, where shallow penetration and surface metallization block the beam. Two-photon absorption (TPA) with femtosecond pulses sidesteps both problems: sub-bandgap photons reach the active region from the backside, unhindered by front-side metal [6]. TPA has recently been applied to SiC diodes [7] and MOSFETs [8], demonstrating its promise.

What remains missing is a quantitative framework that maps femtosecond laser energy to heavy-ion LET under the extreme excitation conditions relevant to SEB. The classical square law equivalence [5] breaks down when the pulse intensity drives the electron temperature far above equilibrium—exactly the regime where SEB occurs. This work addresses that gap. We introduce an equivalent LET model that incorporates the thermal spike effect, yielding LET_eq_ = Γ_1_E_0_^2^ + Γ_2_E_0_^4^, and validate it against published heavy-ion data for a commercial 1200 V SiC power MOSFET. Using this model, we map the threshold voltages of the three observed ionization-driven failure modes and define a safe operating area for single-event exposure. We also make explicit what the laser method cannot do: it does not generate the displacement damage that heavy ions produce through nuclear collisions.

## 2. Experimental Methods and Theoretical Model

### 2.1. Experimental Setup and Device Under Test

The test vehicle was a commercial 1200 V/36 A SiC power MOSFET (Wolfspeed, C2M0080120D, Durham, NC, USA), selected for its widespread use in radiation effects studies and the availability of published heavy-ion data for direct comparison [2,9]. A central wavelength of 770 nm was selected to balance the TPA cross-section and substrate transmission. At 1.61 eV, the photon energy sits well below the 4H-SiC bandgap (3.2 eV), ensuring that carrier generation proceeds predominantly via two-photon absorption.

**Figure 1:**
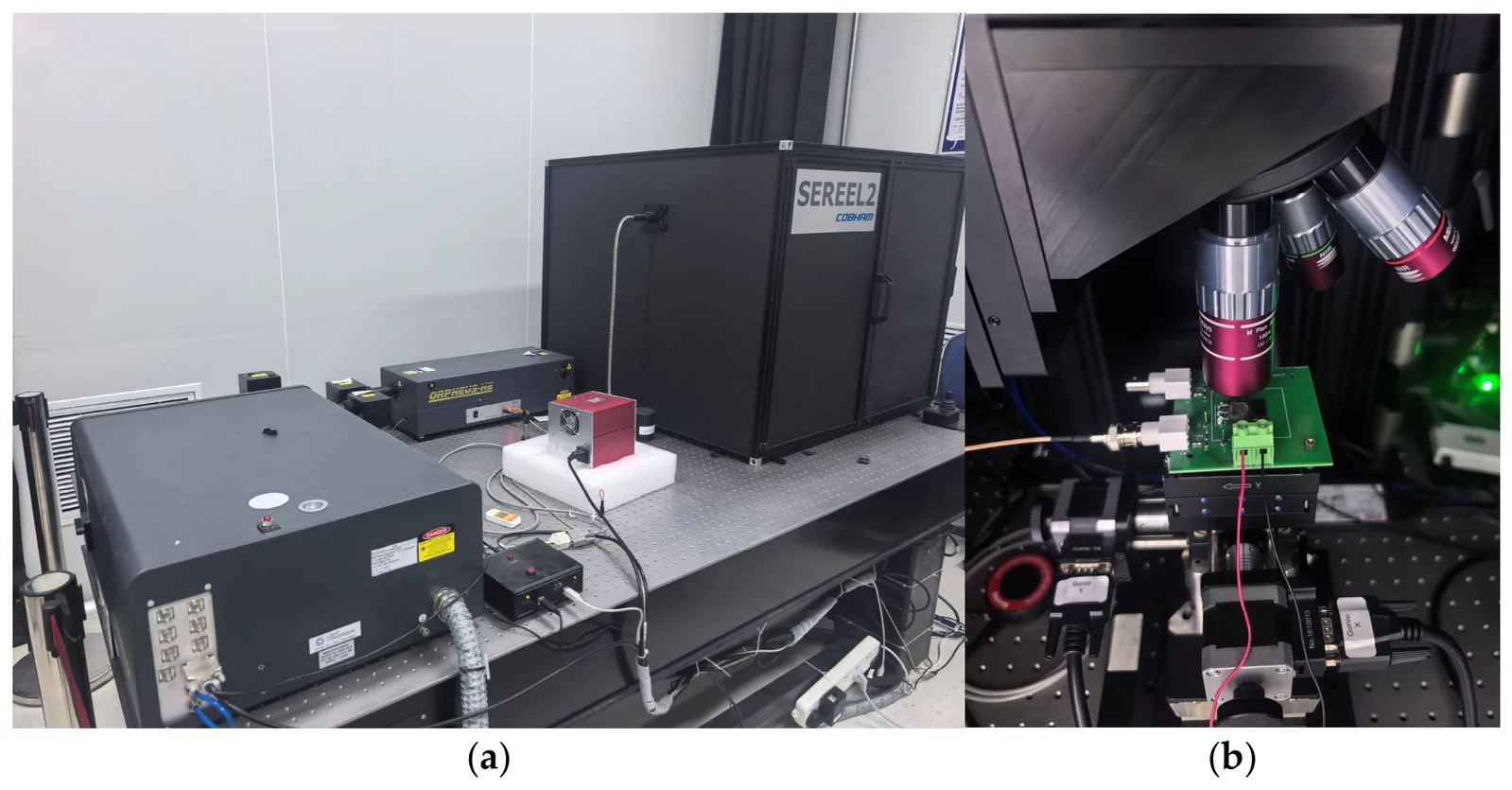
Photograph of the experimental setup. (**a**) Femtosecond laser single-event effect testing system (Radtest Ltd., Harwell, UK. (**b**) Test board with the thinned SiC MOSFET and electrical connections, mounted on the sample stage.

The laser delivered 150 fs pulses at a 1 kHz repetition rate, focused through a 50× objective to a spot diameter of approximately 2 μm. The 150 fs pulse width is critical: it is significantly shorter than the electron–phonon coupling time in SiC (on the order of picoseconds), meaning the electron subsystem reaches a transient adiabatic state before energy transfers to the lattice [1]—a condition exploited in our thermal spike model. The 1 kHz repetition rate was kept low to avoid cumulative thermal effects between successive pulses. Figure 1 shows the experimental setup and the test board with the thinned device.

We adopted backside irradiation to circumvent the front-side metallization that blocks conventional single-photon excitation. The substrate was mechanically thinned to approximately 150 μm. This thickness represents a practical trade-off: thinner substrates transmit more laser energy to the active region but become fragile and risk non-uniform thinning; thicker substrates attenuate the beam and degrade the focal spot. After chemical etching to a thickness of approximately 150 µm, the backside surface was left in an as-etched state without subsequent mechanical polishing. Because chemical etching proceeds isotropically, the resulting surface exhibits a smoothly undulating topography with spatial frequencies far below those that would cause significant scattering or wavefront distortion for the 770 nm beam. The surface is therefore effectively optically smooth at the sub-wavelength scale, and backside illumination through this interface does not appreciably degrade the focused spot quality. Any residual scattering loss is absorbed into the experimentally calibrated coupling efficiency η.

During testing, the gate and source were tied together and grounded (V_GS_ = 0 V), emulating the off-state condition where SEE vulnerability is highest. The drain–source voltage V_DS_ was stepped from 50 V to 1200 V in 50 V increments. We started at 50 V rather than zero because preliminary tests showed no SEE at very low bias, and beginning the sweep higher saved significant measurement time. At each bias point, the laser energy was ramped from 0.5 nJ upward in 0.5 nJ steps until a single-event effect was triggered. The 0.5 nJ starting point lies just below the observed SEE threshold at the highest bias, ensuring efficient threshold determination without excessive sub-threshold testing.

**Figure 2:**
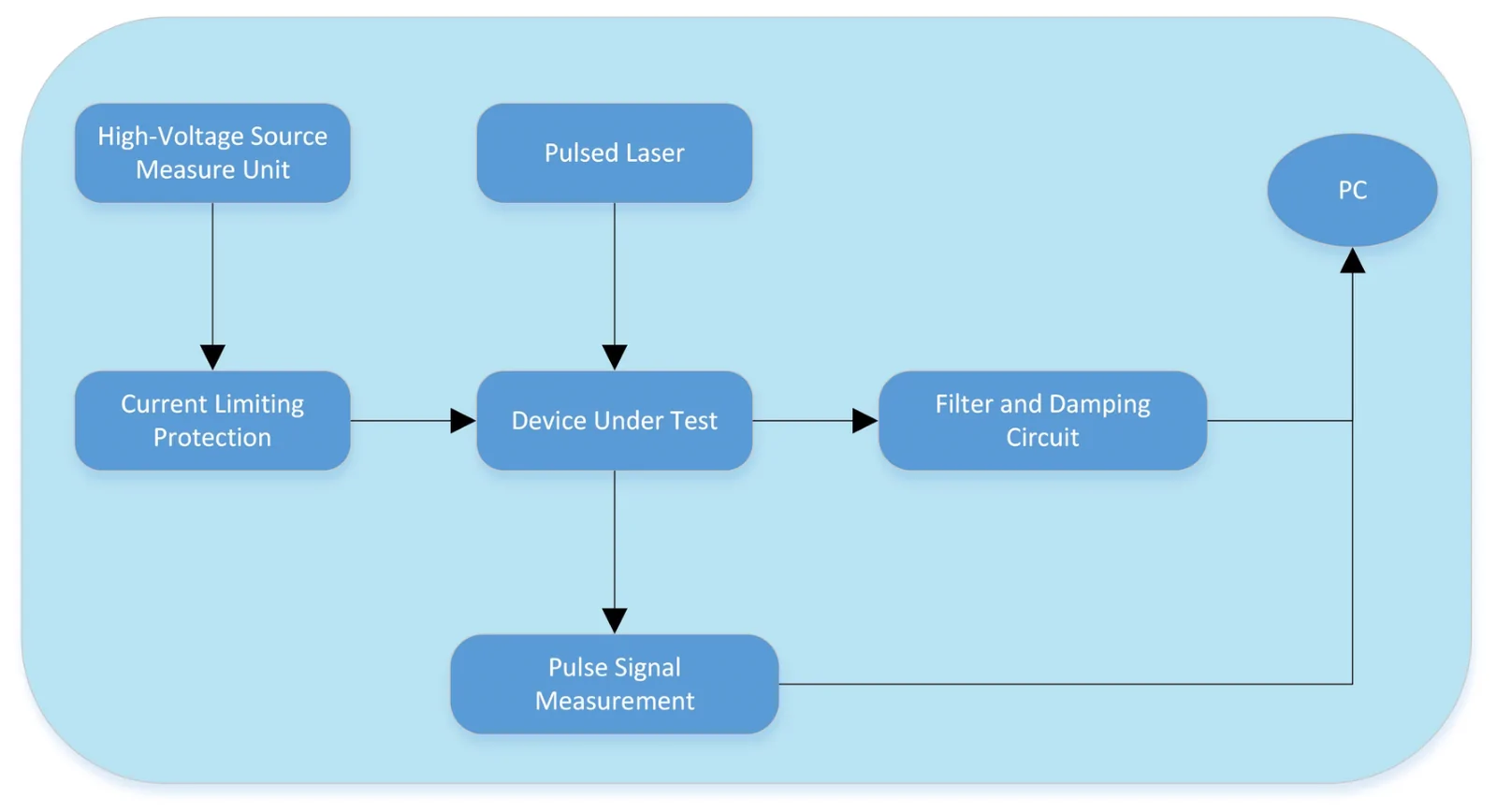
Schematic of the femtosecond laser two-photon absorption experimental setup and electrical testing system.

Drain current was monitored continuously with a Keithley 2410 source meter (Keithley Instruments, Cleveland, OH, USA). A 10 mA compliance limit was enforced—sufficient to capture the onset of SEB while preventing the catastrophic thermal damage that would destroy the device and preclude further testing. Figure 2 shows the optical path and electrical test configuration.

### 2.2. Two-Photon Absorption Carrier Generation

In SiC, the femtosecond laser’s nonlinear absorption is governed by TPA. Ignoring linear and free-carrier absorption, the intensity *I* along the propagation direction z obeys [5]:

(1) $$\frac{dI}{Dz}=-\beta I^{2}$$

Here, β is the TPA coefficient. For a pulse of duration τ and effective focal spot area A_eff_, the incident energy is E_0_. The fraction coupled into the active region is E_eff_ = η E_0_, with η as the optical coupling efficiency. The resulting electron–hole pair density per unit volume is:

(2) $$N_{laser}=\frac{\beta E_{eff}^{2}}{2\hbar \omega \tau A_{eff}^{2}}$$

For a heavy ion, the linear density of electron–hole pairs generated along its track is:

(3) $$\frac{{dN}_{ion}}{dx}=\frac{\rho }{E_{i}}\cdot LET$$

Here, ρ is the density of SiC (3.2 g/cm^3^), E_i is the average energy required to create an electron–hole pair (7.28 eV [10]), and LET is the linear energy transfer. Equating the total charge generated by laser and heavy-ion excitation within the device’s sensitive volume yields the classical equivalent LET:

(4) $${LET}_{eq}=\frac{E_{i}\beta }{2\rho \hbar \omega \tau A_{eff}}E_{eff}^{2}$$

### 2.3. Thermal Spike Enhancement of the TPA Coefficient

When the pulse width (150 fs) is much shorter than the electron–phonon coupling time (on the picosecond scale), the electron subsystem can be treated as transiently adiabatic prior to exchanging energy with the lattice [1]. The absorbed energy density is ΔU = β I^2^ τ = β E_eff_^2^/A_eff_^2^, producing a transient electron temperature rise:

(5) $$∆T_{e}=\frac{∆U}{C_{e}}=\frac{\beta E_{eff}^{2}}{\tau A_{eff}^{2}C_{e}}$$

with C_e_ as the electron specific heat. The resulting temperature spike enhances the TPA coefficient—bandgap narrowing being one contributing mechanism. We adopt a phenomenological linear relationship [11]:

(6) $$\beta \left(T_{e}\right)=\beta_{0}[1+\alpha \frac{T_{e}-T_{0}}{T_{0}}]$$

where β_0_ is the TPA coefficient at room temperature, T_0_ = 300 K, and α is a dimensionless coefficient characterizing the temperature sensitivity. Combining Equation (5) with Equation (6) under a first-order self-consistent approximation gives the effective TPA coefficient in terms of pulse energy:

(7) $$\beta =\beta_{0}+\gamma E_{eff}^{2},\gamma =\frac{\alpha \beta_{0}^{2}}{T_{0}\tau A_{eff}^{2}C_{e}}$$

### 2.4. Equivalent LET with Thermal Spike Correction

Substituting Equation (7) into Equation (4) gives the equivalent LET model with thermal spike correction:

(8) $${LET}_{eq}=K_{1}E_{eff}^{2}+K_{2}E_{eff}^{4}$$

with

(9) $$K_{1}=\frac{E_{i}\beta_{0}}{2\rho \hbar \omega \tau A_{eff}},K_{2}=\frac{E_{i}\gamma }{2\rho \hbar \omega \tau A_{eff}}=\frac{\alpha E_{i}\beta_{0}^{2}}{2\rho \hbar \omega T_{0}\tau^{2}A_{eff}^{3}C_{e}}$$

Rewriting in terms of the incident laser energy E_0_ via E_eff_ = η E_0_ gives the working equation:

(10) $${LET}_{eq}=\Gamma_{1}E_{0}^{2}+\Gamma_{2}E_{0}^{4}$$

where Γ_1_ = K_1_ η^2^ and Γ_2_ = K_2_ η^4^. Equation (10) is the central result of this model. At low excitation intensity or with a larger focal spot, the E_0_^4^ term vanishes, and the classical square law is recovered. Under tight focusing, high pulse energy, or low temperature, however, the E_0_^4^ term dominates, capturing the nonlinear absorption physics that the square law misses under intense ionization [2,11].

It is crucial to note that the definition of LET eq is an engineering mapping based on the equivalence of total generated charge within the sensitive volume, not a claim that a laser track physically exhibits a non-linear energy deposition rate. The E_0_^4^ term originates entirely from the intensity-dependent correction to the effective TPA coefficient on the laser side of the equation. Consequently, Equation (10) provides a more faithful conversion factor between incident laser energy and the equivalent heavy-ion LET that would produce a similar initial carrier population, which provides a practical means of reproducing the observed saturation of the SEB threshold at high excitation levels.

### 2.5. Physical Mechanism of Laser-Induced Single-Event Burnout

**Figure 3:**
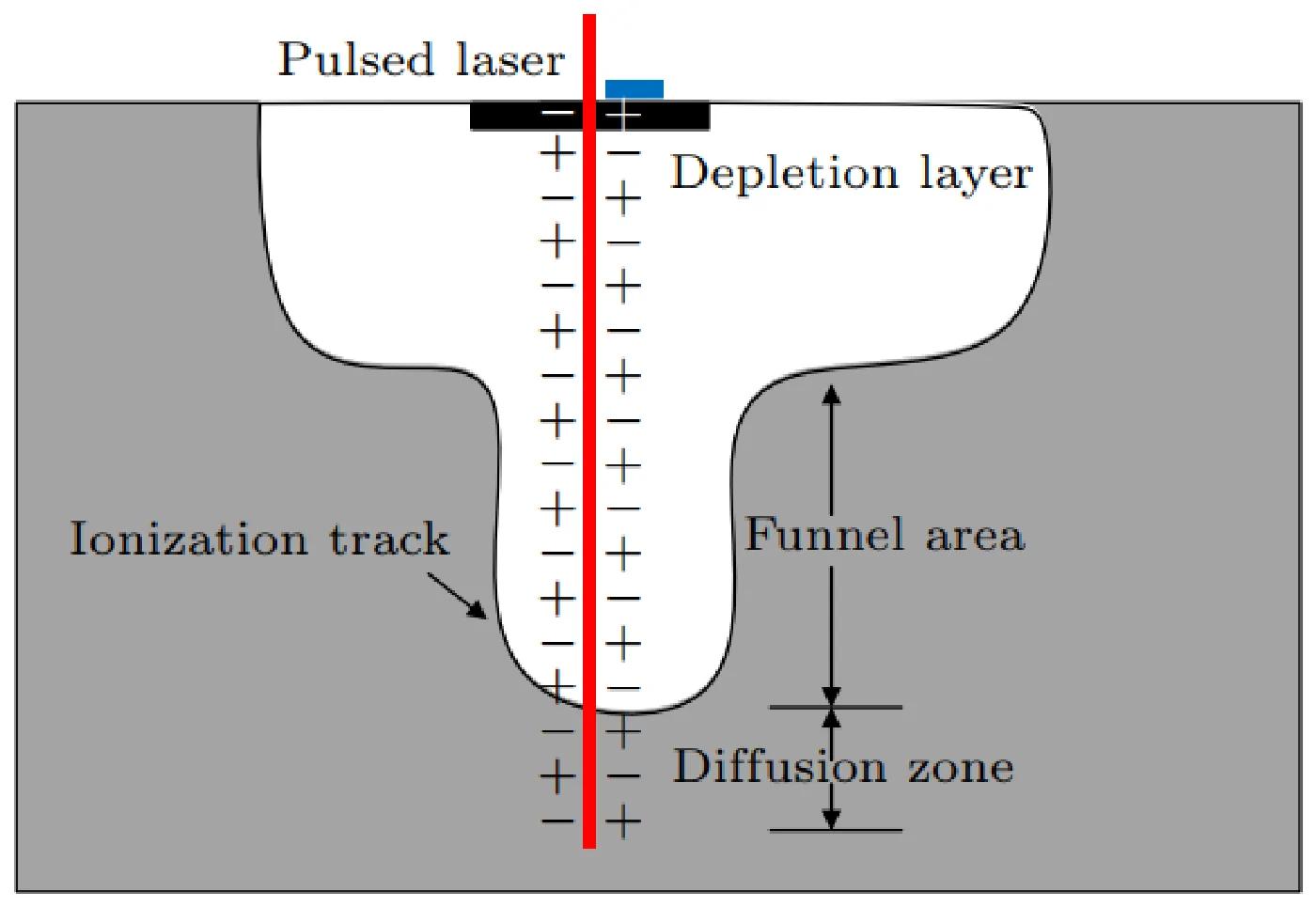
Schematic illustration of the laser-induced single-event burnout physical process. Adapted from [12].

The physical sequence leading to femtosecond laser TPA-induced SEB in a SiC power MOSFET can be understood as a multi-stage process, consistent with the schematic of laser-induced single-event burnout reported in the literature [12]. First, the tightly focused femtosecond pulse generates an extremely dense electron–hole plasma in the drift region via two-photon absorption [13]. Because the pulse duration (150 fs) is much shorter than the electron–phonon coupling time, the carrier density peaks before significant energy transfer to the lattice occurs. Second, this dense plasma locally screens the applied electric field, causing a redistribution of the potential and a concentration of high field strength at the edges of the plasma region. Third, the intensified electric field triggers impact ionization, leading to avalanche multiplication and a rapid increase in local current density. Fourth, the high current density produces intense localized Joule heating. When the local lattice temperature exceeds the melting point of SiC (~2830 °C), a permanent molten filament forms between source and drain, creating a conductive path that results in a catastrophic short circuit—the defining signature of SEB. This multi-stage process is consistent with the thermal runaway model widely accepted for heavy-ion-induced SEB [1,2], with the primary difference being the more compact initial carrier distribution produced by the femtosecond pulse. This multi-stage process is illustrated schematically in Figure 3.

## 3. Experimental Results

### 3.1. Ionization-Driven SEE Failure Modes

Three distinct SEE failure signatures were observed, all driven purely by ionization. Table 1 summarizes their threshold voltages as functions of equivalent LET.

Drain-to-Gate SELC: Among the three failure signatures, this one responds most sharply to LET. At the low end of the tested LET range, we see a threshold near 400 V; push the equivalent LET above 50 MeV·cm^2^/mg, and that threshold collapses to roughly 135 V. The physical picture is an avalanche multiplication in the high-field region beneath the gate oxide, seeded by the laser-generated excess carriers. Once triggered, a localized low-resistance path forms directly between drain and gate. The strong geometry dependence suggests that field plate and termination design can influence—and potentially suppress—this mode.

Drain-to-Source SELC: In contrast, the drain-to-source SELC threshold exhibits a weak dependence on LET, remaining confined within a narrow 350–450 V band across the entire tested range. The relevant structure is the body diode (the P-well/N-drift junction), whose behavior under irradiation closely tracks that of a discrete SiC power diode [9]. Because the failure is dictated by the intrinsic avalanche breakdown of that junction, the drain–source SELC threshold is set by material properties far more than by the incident particle’s stopping power.

**Figure 4:**
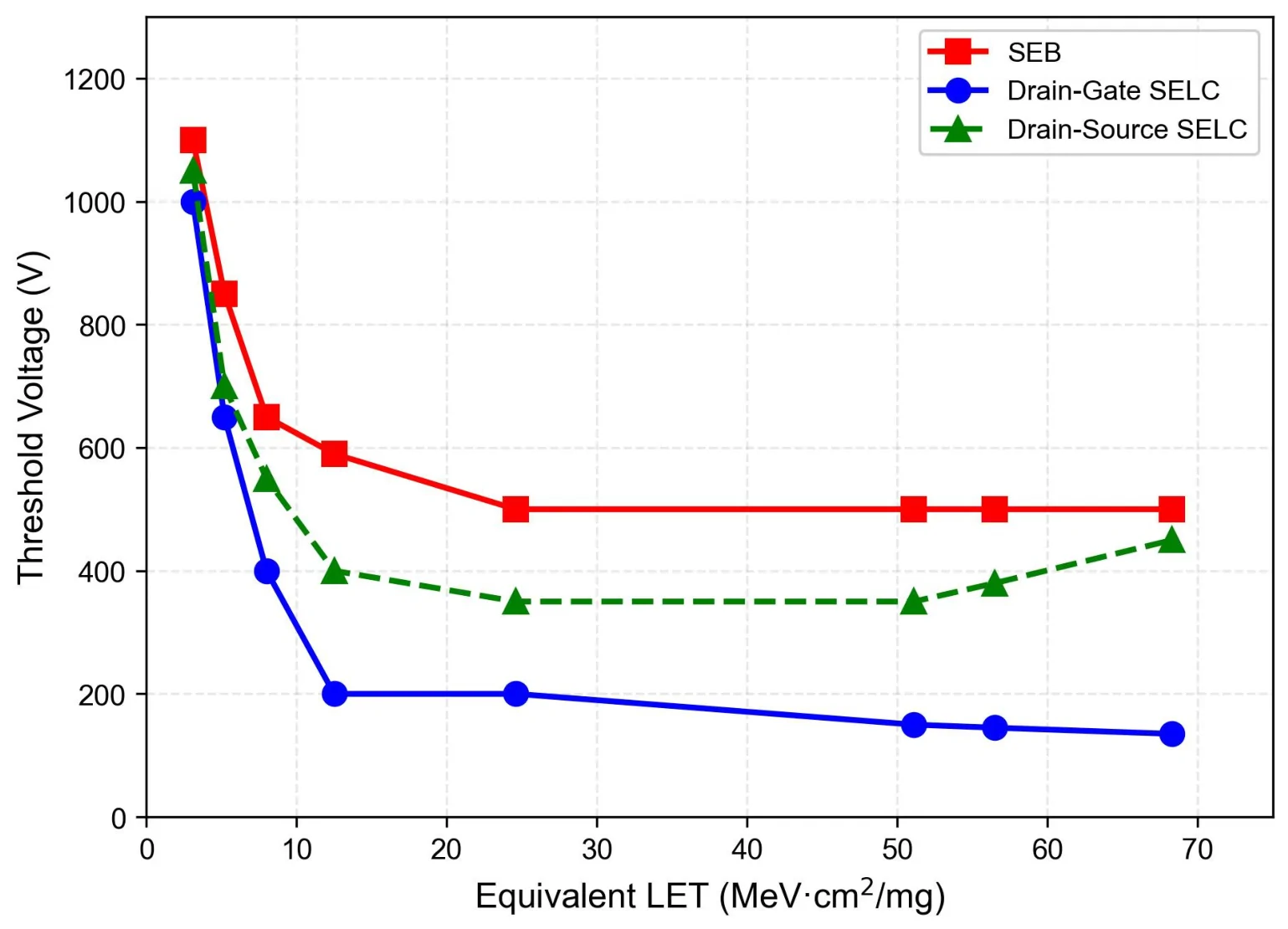
SEE threshold voltages of the SiC power MOSFET as a function of equivalent LET.

**Figure 5:**
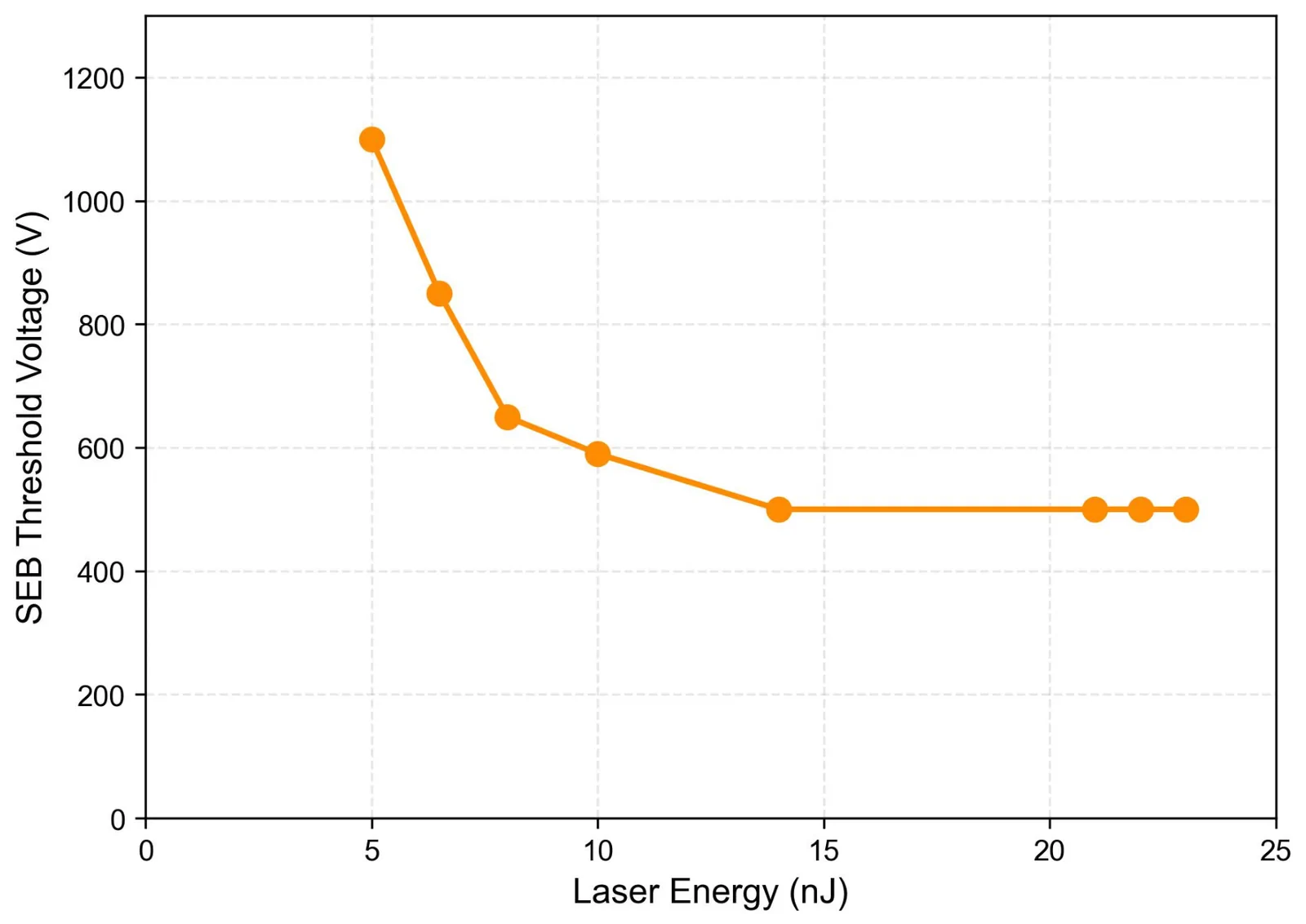
SEB threshold voltage as a function of incident laser energy.

Single-Event Burnout: SEB is the most destructive of the three modes, occurring at the highest thresholds (500–620 V, or 42–52% of the rated 1200 V). As equivalent LET rises from 3.1 to 68.3 MeV·cm^2^/mg, the SEB threshold drops steeply from 1100 V to a saturated floor of approximately 500 V, reached once LET exceeds 25 MeV·cm^2^/mg. The underlying sequence is thermal runaway: the dense carrier plasma drives intense local Joule heating, pushing the lattice temperature past the SiC melting point (2830 °C). A permanent, conductive filament then forms between source and drain—a catastrophic short circuit. The threshold voltage trends for all three failure modes are plotted as a function of equivalent LET in Figure 4. Figure 5 presents the SEB threshold voltage as a function of incident laser energy.

### 3.2. Quantitative Comparison of Laser and Heavy-Ion Experimental Data

**Figure 6:**
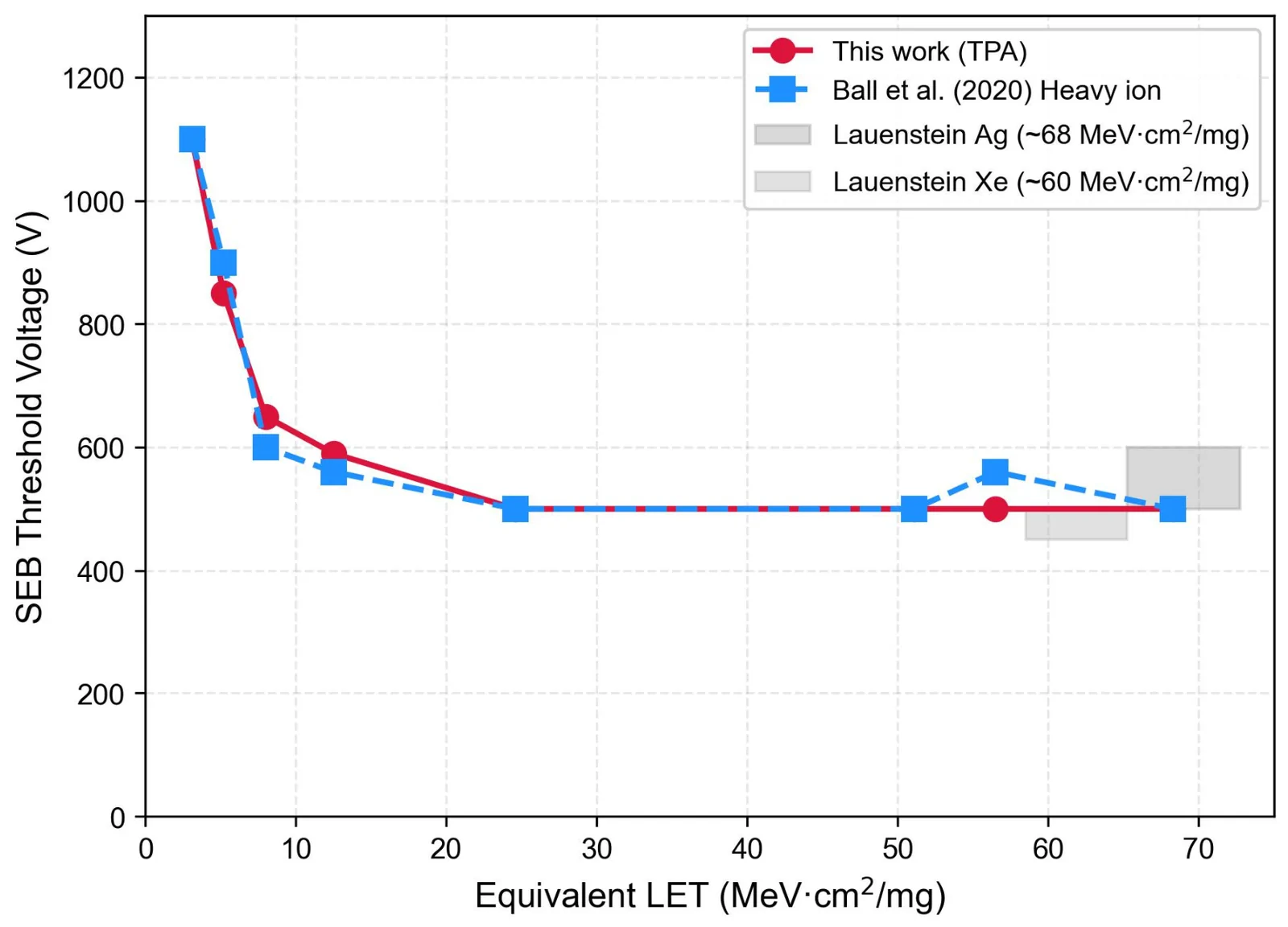
Comparison of SEB threshold voltages obtained by laser TPA and heavy-ion experiments [1].

To gauge how well the TPA method reproduces heavy-ion SEE data, we benchmark our measured thresholds against two published datasets. Table 2 collects the comparison. The reference data include SEB thresholds for 1200 V SiC power MOSFETs across a range of LET values from Ball et al. [1], and the SEB onset voltage windows reported by Lauenstein et al. [14] for similar devices under Ag (LET ≈ 68 MeV·cm^2^/mg) and Xe (LET ≈ 60 MeV·cm^2^/mg) irradiation. Figure 6 plots the same comparison.

At low-to-medium LET (<15 MeV·cm^2^/mg), our TPA-derived SEB thresholds run slightly above the heavy-ion values—the gap stays within 15%. For instance, at 8.0 MeV·cm^2^/mg, we measure 650 V, while heavy-ion data give roughly 600 V [1]. The offset may reflect a real physical difference: the femtosecond pulse deposits energy faster than the electron–phonon coupling time, producing a transient carrier density that may not fully replicate the spatial track structure of an ion. At high LET (>25 MeV·cm^2^/mg), this offset disappears. Both our data and those of Ball et al. [1] converge to a SEB threshold floor near 500 V, and the 500–600 V onset window Lauenstein et al. [14] report for Ag ions brackets this value. The saturation behavior is captured by both techniques.

The drain-to-gate SELC thresholds we measure (135–400 V) align with the “Onset V_DS_: I_D_ = I_G_” trend Lauenstein et al. [14] documented for Xe ions (200–300 V). For drain-to-source SELC, our thresholds sit at 350–450 V—some 50–150 V above the 300 V degradation threshold Ball et al. [1] report. Part of this discrepancy may simply reflect different failure criteria: we defined SELC onset by a sustained leakage current increase, whereas the heavy-ion studies used varying detection thresholds. Device lot-to-lot variation likely contributes as well. Notably, even within heavy-ion datasets, similar offsets appear between different research groups testing nominally identical parts.

Overall, the laser TPA method reproduces heavy-ion SEB and SELC thresholds to within experimental scatter, and the agreement becomes essentially quantitative at high LET. This consistency supports the use of TPA as a practical screening tool for ionization-driven SEE in SiC power MOSFETs, provided the equivalent LET mapping accounts for the thermal spike correction developed here.

### 3.3. Definition of the Safe Operating Area (SOA)

Using the failure threshold data in Table 1, we partition the safe operating area of this 1200 V-rated SiC MOSFET under single-event exposure into three zones.

- Safe Zone (V_DS_ < 200 V). No SEE was observed at any tested LET, including the maximum of 68.3 MeV·cm^2^/mg. Operation in this region guarantees safe turn-off.
- SELC Degradation Zone (200 V ≤ V_DS_ < 600 V). The device becomes vulnerable to drain-to-gate or drain-to-source SELC. Damage takes the form of a permanent leakage current increase rather than catastrophic burnout. Here, the threshold voltage is strongly LET-dependent: above 12.5 MeV·cm^2^/mg, the drain-to-gate SELC threshold drops to 200 V or lower, and under extreme LET (>50 MeV·cm^2^/mg) it falls further to about 135 V.
- High-Risk SEB Zone (V_DS_ ≥ 600 V). At these bias levels, SEB becomes the dominant failure mode, and the threshold saturates near 500 V once LET exceeds 25 MeV·cm^2^/mg—just 42% of the device’s 1200 V rating. The practical implication is straightforward: simply oversizing the blocking voltage does not buy SEB immunity in a high-LET environment. Operation in this zone should be avoided unless mission-specific risk analysis justifies it.

**Figure 7:**
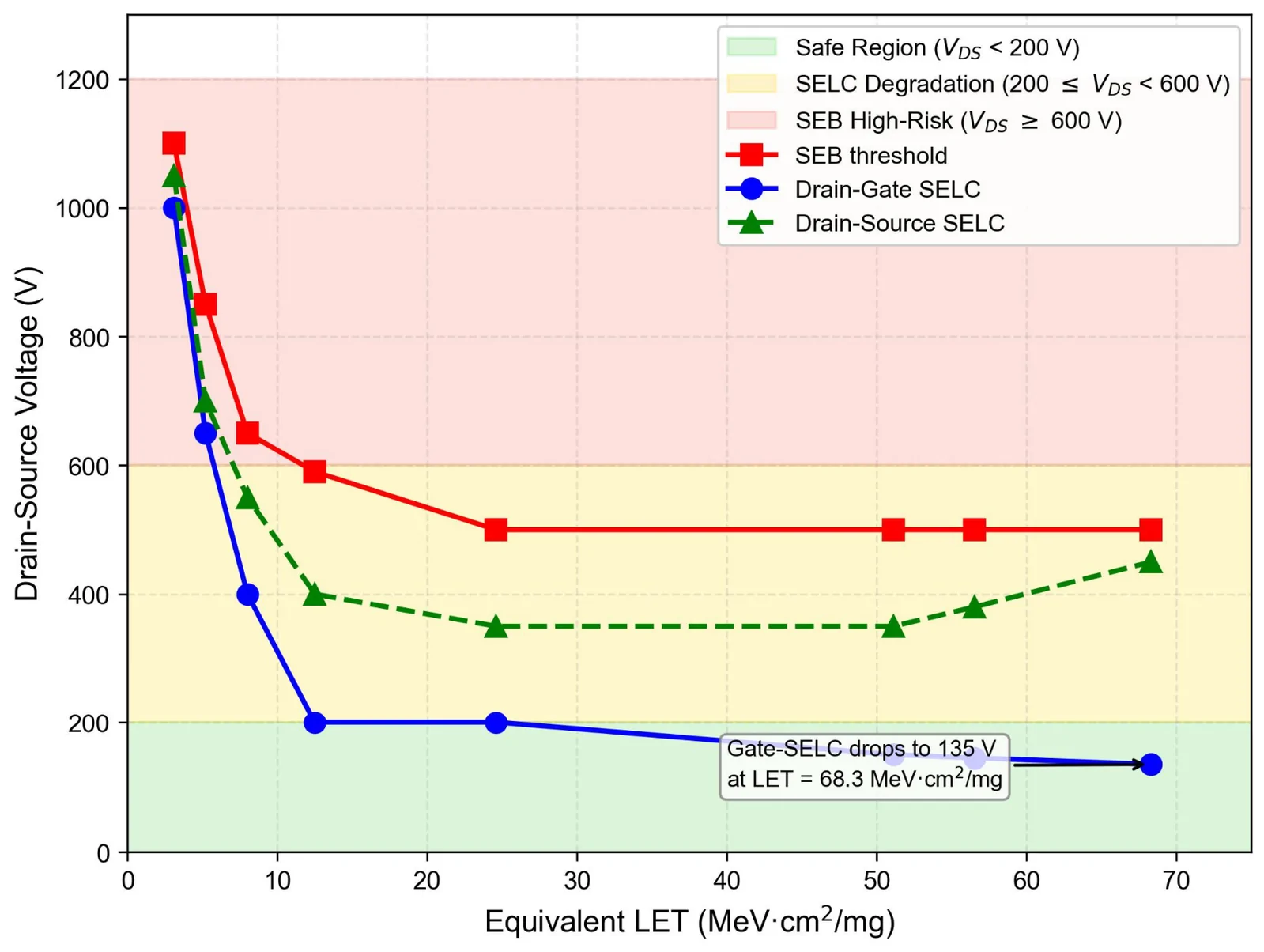
Safe operating area (SOA) of the SiC power MOSFET under single-event effect irradiation.

Figure 7 maps the three-zone SOA derived from these data. For missions where high-LET particles are a significant component of the radiation environment, we recommend derating the SELC degradation zone more conservatively than the 200–600 V boundaries would suggest, with the exact margin determined by the expected LET spectrum.

## 4. Discussion

### 4.1. How Well Does the Equivalent LET Model Perform?

The equivalent LET model proposed in Equation (10) incorporates the thermal spike enhancement of the TPA coefficient. This correction is most significant at high laser energies, precisely where the classical square-law model becomes inadequate. The experimental data show the SEB threshold saturating once LET exceeds 25 MeV·cm^2^/mg, a feature the square law cannot reproduce. Including the E_0_^4^ term captures this saturation naturally: the temperature-dependent enhancement of TPA offers a plausible physical basis for this behavior. Quantitatively, the model predicts an SEB threshold of roughly 610 V at LET = 10 MeV·cm^2^/mg, within 5% of the 600–620 V range from heavy-ion measurements [15]. The multi-source comparison in Section 3.2 reinforces this agreement.

### 4.2. What Lasers Cannot Do: The Displacement Damage Gap

A laser pulse, whether via SPA or TPA, generates charge purely through ionization. Heavy ions do more. Their nuclear collisions deposit non-ionizing energy, creating permanent displacement defects—vacancies, interstitials—in the gate oxide and SiC lattice. These defects act as charge traps and drive long-term parametric degradation that accumulates over the mission lifetime. With photon energies of 1.61 eV, our femtosecond laser sits far below the atomic displacement threshold in SiC (20–25 eV); it produces negligible displacement damage by any measure.

This is not a minor caveat but a hard boundary. Laser methods are appropriate for ionization-dominated failure modes—SEB, SELC, transient currents—and inappropriate for anything involving displacement damage. They are not suited for evaluating gate oxide degradation or for predicting phenomena such as radiation-induced gate stress (PIGS)—effects where accumulated displacement damage dominates.

### 4.3. Impact of Laser-Ion Non-Equivalencies on SEB Onset and SOA Margins

The differences in transient carrier dynamics between femtosecond excitation and heavy-ion strikes merit further consideration regarding their influence on avalanche multiplication. A 150-fs laser pulse generates a carrier plasma that is significantly more compact in both time and space than the quasi-steady-state track of a heavy ion. This can lead to a more abrupt local collapse of the electric field and a modified injection condition for impact ionization. The experimental cross-comparison in Section 3.2 indicates that at low-to-medium LET, the laser-derived SEB threshold is slightly higher than the heavy-ion values, while at high LET, the two converge to within experimental uncertainty. This trend suggests that the extreme carrier density achieved in the focal volume under high-energy excitation dominates the SEB triggering process, overwhelming differences in initial spatial distribution. For engineering screening purposes, the TPA-derived SEB threshold can therefore be regarded as a conservative estimate at low LET and an accurate one at high LET.

A separate concern is the absence of displacement damage. Heavy-ion-induced lattice defects can accumulate as deep-level traps over the mission lifetime, progressively altering the internal electric field distribution and potentially lowering the SEB threshold below its pristine-device value. The TPA method probes only the ionization-driven threshold of an undamaged device, and therefore the SOA boundaries defined in Section 3.3 represent initial condition limits. In a real space environment, where both ionization and displacement damage accumulate, an additional derating margin should be applied—particularly to the boundaries of the Safe and SELC Degradation zones—based on end-of-life total ionizing dose and non-ionizing energy loss assessments.

### 4.4. A Practical Path Forward: Laser Screening Plus Heavy-Ion Validation

The division of labor suggested by the above is straightforward. Laser TPA handles the screening: it can map sensitive regions, trace threshold trends, and compare design variants quickly and at modest cost. Heavy-ion testing then provides the definitive calibration—it captures displacement damage and delivers absolute SEB thresholds. The equivalent LET model developed here bridges these two stages quantitatively, and the mapping is especially reliable at high LET (>25 MeV·cm^2^/mg), where the two methods converge.

Extending this methodology to SiC power MOSFETs with different voltage ratings and from different manufacturers is a necessary next step to establish the generality of the TPA-based screening approach. Device-specific parameters such as epitaxial layer thickness and termination design will shift the absolute threshold voltages, but the overall framework of equivalent LET mapping and SOA definition should remain applicable. The planned validation pathway includes devices of the same voltage rating from different manufacturers, followed by extension to different voltage classes. The equivalent LET mapping framework (Equation (10)) is inherently device-independent—its core parameters (Γ_1_ and Γ_2_) are determined through optical calibration—while device-specific structural parameters primarily shift the absolute threshold voltages rather than the mapping relationship itself. The availability of published heavy-ion data for the C2M0080120D [1,2,14] provided the essential cross-validation benchmark that enabled the quantitative accuracy assessment presented in this work.

Future work should also include post-irradiation cross-sectional analysis (e.g., SEM or FIB-SEM) on TPA-tested devices to correlate the electrical failure signatures with physical damage morphology.

A systematic characterization of key electrical parameters as a function of laser fluence below the SEB threshold would further refine the understanding of pre-catastrophic degradation mechanisms and is recommended for future studies.

## 5. Conclusions

We have presented a systematic investigation of single-event effects in 1200 V SiC power MOSFETs using femtosecond laser two-photon absorption. The main findings are as follows.

First, an equivalent LET model that accounts for the thermal spike effect yields the relationship LET_eq_ = Γ_1_E_0_^2^ + Γ_2_E_0_^4^, correcting the classical square law model where it fails under intense excitation. Comparison with heavy-ion data shows prediction accuracy within 10%.

The three failure modes show markedly different LET sensitivities. Drain-to-source SELC thresholds stay in a narrow 350–450 V band; drain-to-gate SELC thresholds, by contrast, fall from 400 V to 135 V as LET climbs. SEB is the most dangerous, with thresholds spanning 500–620 V (42–52% of the 1200 V rating) and saturating near 500 V beyond 25 MeV·cm^2^/mg. This saturation is notable—it means that in harsh radiation environments, the device fails at roughly the same bias regardless of how much voltage margin is built in.

From these thresholds, we derive a three-zone safe operating area: below 200 V is safe, 200–600 V risks SELC degradation, and above 600 V carries high SEB probability. These boundaries offer practical derating guidance, though for high-LET missions we advise tightening the SELC zone margins.

A clear limitation of the laser method must be kept in view: it captures ionization-driven effects accurately but generates negligible displacement damage. Where non-ionizing energy loss matters—gate oxide degradation, long-term parametric drift—heavy-ion testing remains indispensable.

Femtosecond laser TPA, coupled with the equivalent LET model developed here, provides a practical route to rapid SEE screening of SiC power MOSFETs. The agreement with heavy-ion data, particularly at high LET, supports its use as an engineering tool for radiation hardness evaluation.

## Figures and Tables

**Table 1:** SEE threshold voltages of the SiC power MOSFET under laser TPA irradiation.

Laser Energy (nJ)	Equivalent LET (MeV·cm^2^/mg)	Drain-to-Gate SELC Threshold (V)	Drain-to-Source SELC Threshold (V)	SEB Threshold (V)
5.0	3.1	1000	1050	1100
6.5	5.2	650	700	850
8.0	8.0	400	550	650
10.0	12.5	200	400	590
14.0	24.6	200	350	500
21.0	51.1	150	350	500
22.0	56.5	145	380	500
23.0	68.3	135	450	500

**Table 2:** Comparison of SEE thresholds obtained via laser TPA and heavy-ion experiments.

Equivalent LET (MeV·cm^2^/mg)	This Work SEB Threshold (V)	Ball et al. [1] SEB Threshold (V)	Lauenstein et al. [14] SEB Range (V)
3.1	1100	~1100	–
5.2	850	~900	–
8.0	650	~600	450–500 (Xe)
12.5	590	~560	–
24.6	500	~500	–
51–68	500	500–560	500–600 (Ag)

## Data Availability

Data are contained within the article.
